# Pacemaker Pocket Erosion: A Critical Issue Requiring Immediate Attention

**DOI:** 10.7759/cureus.75581

**Published:** 2024-12-12

**Authors:** Colin McGuire, Jeniffer Naitore, Vijay Ramu

**Affiliations:** 1 Cardiology, Quillen College of Medicine, East Tennessee State University, Johnson City, USA; 2 Internal Medicine, Johnston Memorial Hospital, Abingdon, USA

**Keywords:** explanted pacemaker, pacemaker complication, pacemaker extraction, pacemaker infection, pacemaker pocket infection

## Abstract

Cardiac implantable electronic devices (CIEDs), including pacemakers, implantable cardiac defibrillators (ICD), and cardiac resynchronization therapy (CRT) devices, regulate heart rate and rhythm in patients with cardiac conditions. With an aging population, CIED-related complications, especially pacemaker pocket infections, are rising. Risk factors include frailty, older age, and superficial device fixation, while risk mitigation involves larger pocket sizes, submuscular fixation, and absorbable antibacterial envelopes. The debate continues regarding the optimal timing for device removal and lead extraction.

This report presents a case of a 77-year-old male with a history of atrial fibrillation and prior methicillin-susceptible Staphylococcus aureus (MSSA) bacteremia, who was admitted with infection symptoms and a pacemaker erosion. Blood and wound cultures confirmed MSSA and the patient underwent successful lead and device extraction. He was treated with daptomycin and discharged two days after admission with close follow-up by infectious disease, cardiology, and wound care specialists.

The case emphasizes the importance of timely intervention in CIED infections, highlighting occult bacteremia where no infection source is identified. Early removal, particularly within one day of presentation, led to a favorable outcome. Simple lead extraction was chosen because the device had been in place for less than a year, and age and comorbidities did not influence the decision.

In the prior MSSA bacteremia episode, early lead and generator extraction might have prevented the second admission, reinforcing the value of early intervention. These findings underscore the need for vigilant monitoring and suggest that future guidelines could benefit from stratifying lead and device removal strategies based on implantation timing to enhance patient outcomes.

## Introduction

With the advent of an increasingly aging population, the use of cardiovascular implantable electronic devices (CIED) is on the rise. Consequently, major complications associated with CIED will concurrently increase. One such complication is pacemaker pocket erosion, a rare but significant issue classified within the overall pocket infection rate of 1.9 per 1,000 device-years [1]. A significant increase in all-cause mortality at 12 months has been noted with major infections [2]. Risk factors for device erosions include frailty, older age, and superficial fixation of the CIED in a subcutaneous pocket [3]. Risk mitigation is not well understood, but expert opinion includes a larger pocket, fixation of the CIED in the submuscular plane, and an absorbable antibacterial envelope [3]. Consensus and practice guidelines have been published on the prevention and management of CIED-related infections, including erosions, with efforts to improve their distribution and implementation. However, significant knowledge gaps and inadequate adherence to these guidelines remain a challenge. Gaps in physicians’ knowledge, skills, and confidence at all stages of CIED care contribute to the inability to mitigate the risks of device infections, including erosions [4]. In addition, most patient risk factors, particularly those related to disease comorbidities, are challenging to modify or are otherwise non-modifiable.

When an erosion occurs resulting in device exposure, a presumptive diagnosis of a CIED infection can be made. While there are rare cases of salvaging the device, it is recommended to remove the device and all leads in addition to obtaining cultures from the pocket, device, and leads (Class I-B) [5,6]. Furthermore, removal of the leads at an earlier stage, as opposed to a delayed approach, has been shown to result in lower rates of in-hospital morbidity and one-year all-cause mortality [7]. There has been some variation in the definition of early versus delayed extraction but all seem to emphasize the same concept. Definitions include extraction within 10 days after indication versus >10 days after indication, extraction at initial presentation versus after failure of antibiotics, and <7 days from hospital admission to extraction versus >7 days [8].

There, however, has been no clear delineation for when simple versus complex lead removal must be applied in CIED pocket erosion settings. Simple extraction is generally considered effective for leads implanted within one to two years, while complex techniques are often required for longer dwell times [9]. However, studies have demonstrated success with simple extraction even more than two years after device implantation [4,10]. The purpose of this case report is to highlight the importance of early, simple lead removal in cases of CIED pocket erosion. Additionally, we emphasize a specific type of CIED infection, occult bacteremia with probable CIED involvement, where no other source of infection is identified, for which CIED removal is recommended, depending on the isolated organism.

## Case presentation

The patient is a 77-year-old male with prior medical history of paroxysmal atrial fibrillation, diurnal symptomatic bradycardia occurring over three months, non-obstructive coronary artery disease, pulmonary fibrosis requiring oxygen, hypertension, hyperlipidemia, former tobacco use disorder, and spinal stenosis. He had a pacemaker (Dual-Chamber Biotronik, Boston Scientific, Edora ProMRI) placed eight months prior to his current presentation for tachycardia-bradycardia syndrome. Notably, the patient had concerns about methicillin-susceptible staphylococcus aureus (MSSA) bacteremia two months before the current presentation where no source of infection was identified for which he was treated with intravenous antibiotics. A transesophageal echocardiogram (TEE) was done and then showed no evidence of bacterial endocarditis.

**Figure 1:**
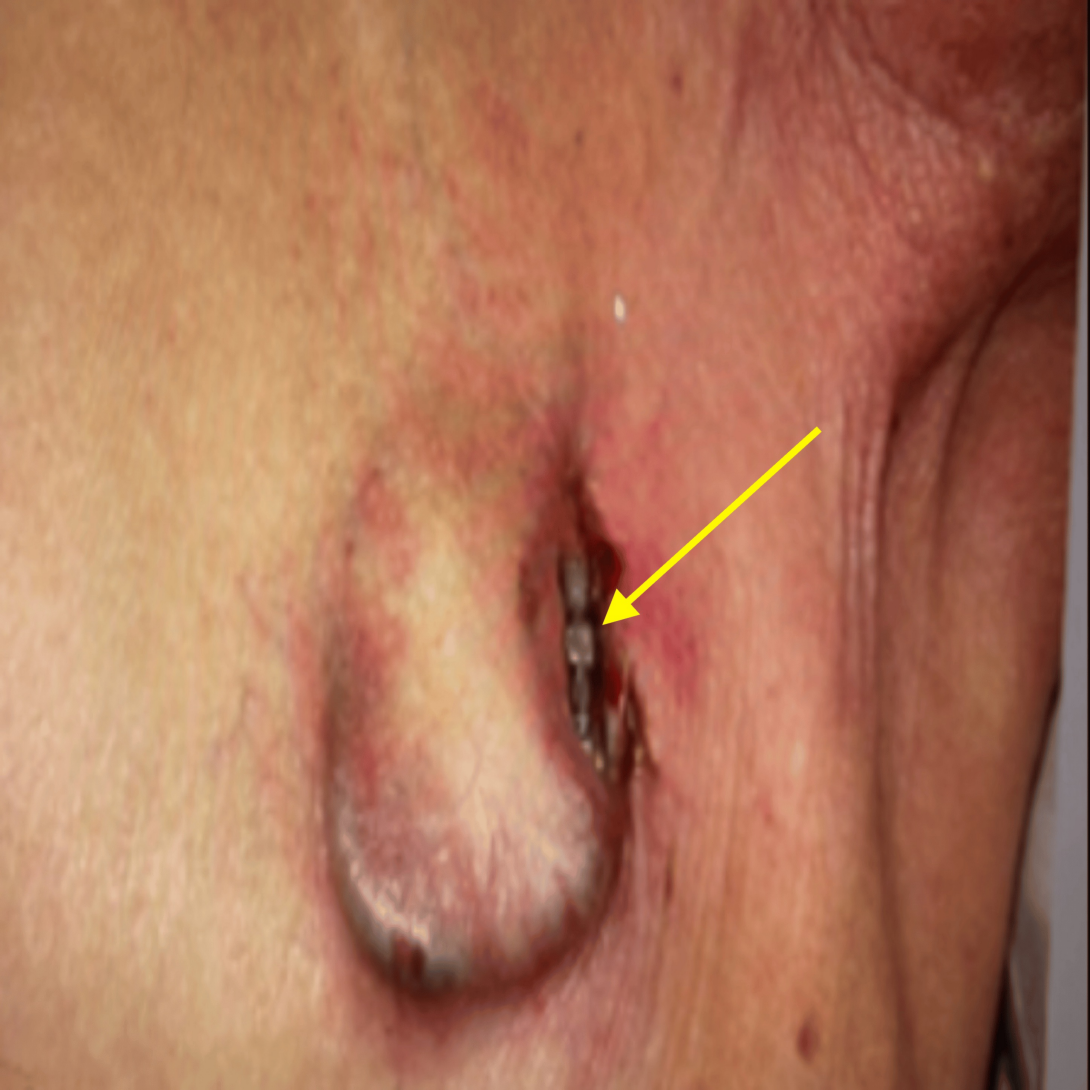
Pacemaker erosion before explantation The arrow points toward the exposed pacemaker.

For this admission, he presented to the emergency department with fever, myalgias, and chills, and on examination, it was noted that the pacemaker site was eroded, and the generator was visible with surrounding areas of purulence, but no tissue necrosis was evident. The surrounding skin was warm and erythematous (Figure 1). Vitals: temperature: 98.9, blood pressure: 101/84, respiratory rate: 22, and pulse: 77. He was noted to have an elevated white blood cell count of 14300 cells/mL and CRP of 126.2 mg/dL. A limited two-dimensional transthoracic echocardiogram showed no obvious evidence of vegetation on the visualized portions of the valves or device leads. Blood cultures at two peripheral sites were obtained, an infectious disease consult was obtained and empiric Vancomycin and Zosyn were ordered for antibiotic coverage, and the patient was admitted for generator and pacer wires extraction the following day. Upon device check, it was determined that the device was functioning appropriately with an atrial impedance of 700 ohms and ventricular impedance of 800 ohms, atrial thresholds of 0.8 V and ventricular threshold of 1.0 V both at 0.4 ms, atrial sensing of 2.1 mV and ventricular sensing of 4.0 mV, and non-pacemaker dependence was demonstrated. Pacemaker settings did not differ from the time of implantation. A decision was made to extract the device and wires without re-implantation at this time, due to the absence of symptoms and no indication of pacemaker dependence.

**Figure 2:**
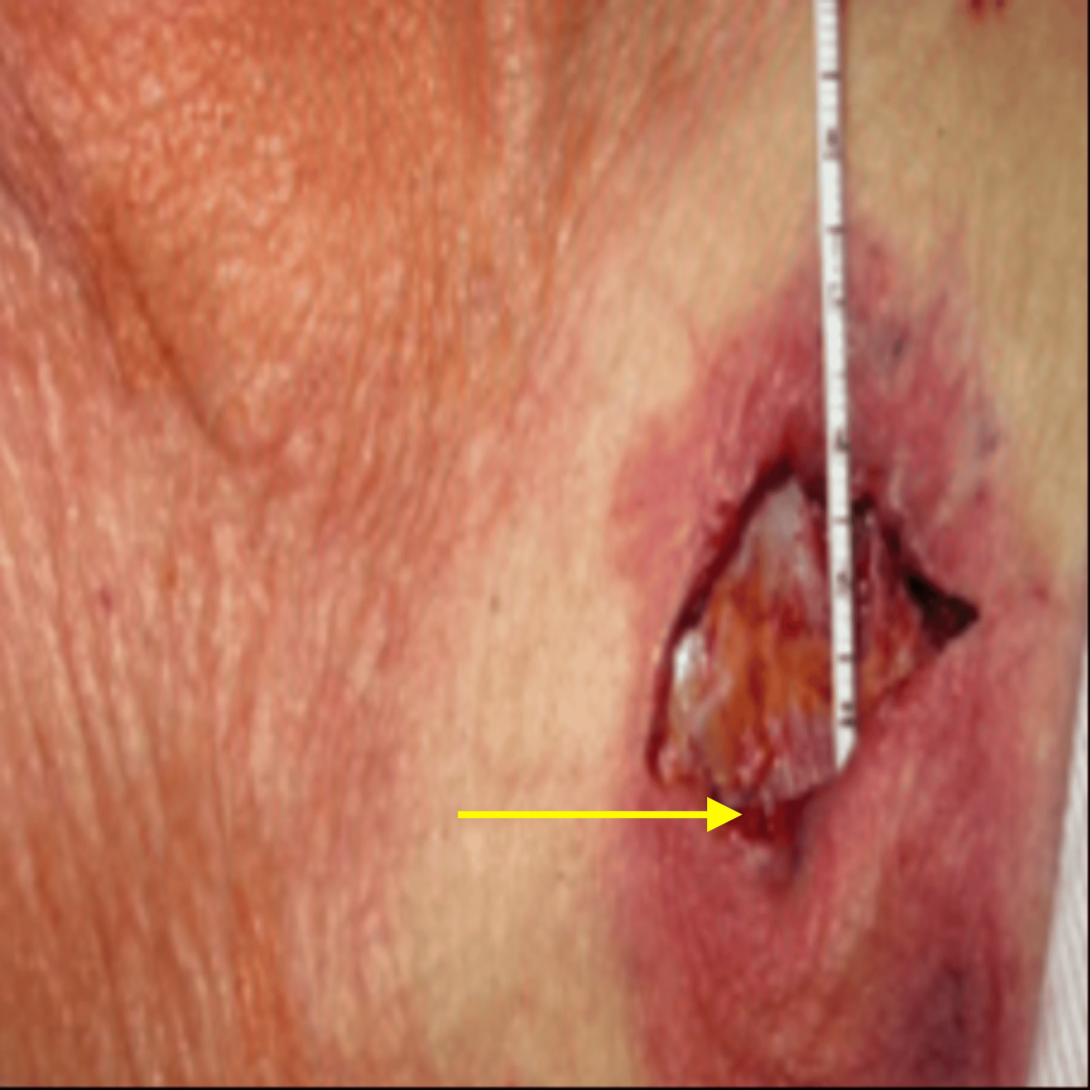
Pacemaker explantation The arrow points toward purulent drainage.

The following day, complete device removal was pursued; it was noted the header was visible with surrounding necrotic tissue and purulent drainage. After a timeout, intravenous fentanyl 2 mcg and intravenous midazolam 50 mg were used for general anesthesia with anesthesia backup. After local lidocaine administration, the skin was incised, and the generator along with the leads was exposed. Purulent fluid was noted surrounding the device. The pulse generator was disconnected from the pacing leads, removed, and sent for culture. Notably, the ventricular lead was not secured to the pre-pectoral fascia and freely mobile through the prior venotomy. The lead anchors were removed, and the helix was retracted for both atrial and ventricular leads under fluoroscopic guidance. Both leads were completely removed from the patient then placed in a sterile container and sent for cultures. The purulent and friable tissues were then dissected away followed by wound irrigation and closure (Figure 2). Cultures from both the blood and the local wound, including the device, grew MSSA. The generator and leads both grew MSSA cultures. A peripherally inserted central catheter was placed, and he was continued with intravenous daptomycin 6 mg/kg/day for six weeks and discharged home two days following a successful procedure with a wound care follow-up and infectious disease follow-up in a week. He followed up after two months in the cardiology clinic after completing a six-week course of antibiotics. He had been feeling well, and the pacemaker generator explant site was healing well. TEE showed no evidence of infective endocarditis. CT-angiogram was done to assess for aortic root abscess and was negative. RA repeat 14-day Holter monitor revealed a 72% atrial fibrillation burden, with 55% of the time showing bradycardia (<60 bpm) and otherwise normal ventricular rates between 60 bpm and 100 bpm, with no patient-triggered events.

## Discussion

CIED-related infection types include superficial infections, isolated pocket infections, isolated pocket erosions (which should be considered infections), bacteremia, pocket infections with bacteremia, CIED-related endocarditis without pocket infection, pocket infections with endocarditis, and occult bacteremia with probable CIED infection where no other source of infection is identified [9]. Early local signs of inflammation may be mild and include erythema, warmth, and fluctuation. Pocket destruction, adherence, or threatened erosion are also signs to look for. Generator or proximal lead exposure should be considered an infection, irrespective of local culture results. Systemic infection without local infection is hard to diagnose. Symptoms may be non-specific and include fever, chills, and night sweats. Embolic phenomena and osteomyelitis can be seen and are important to keep in mind [6]. Furthermore, elevation of impedance values or loss of capture during device check may indicate compromise of the leads.

The incidence of CIED infections is approximately 1.5%. In a cohort of Medicare patients, most of whom are over 65 years of age, CIED infection rates ranged from 1.4% to 2% within the first few months of implantation [11]. Similarly, Nationwide Inpatient Sample (NIS) discharge records from 1993 to 2008 reported an annual incidence of CIED infections at 1.61%. The increased incidence coincided with increasing comorbidities, which tend to increase with age. Overall, the annual rate of CIED infections has been increasing [12].

CIED infections can occur both early and late after implantation [11,13]. A review of CIED infections requiring lead extraction at a European center showed relatively equal rates of infection necessitating extraction: 30.79% within 0-12 months after a CIED-related procedure, 29.89% between 13 and 36 months, and 39.32% more than 36 months after the procedure [13]. Pocket infections have been demonstrated to be the most common early infectious complication, while lead-related infective endocarditis (LRIE) has been shown to be the most common late complication [13].

In our case, the patient presented with a pocket infection (Figure 1) and bacteremia (MSSA isolate identified in both local and blood cultures) approximately eight months after device implantation. In hindsight, this was presumed to be preceded by occult MSSA bacteremia with probable CIED infection, though not proven, two months earlier. The latter was treated with IV antibiotics alone, which may have led to a recurrence of MSSA bacteremia due to the presumed lack of early source control of the pocket infection.

Extraction is recommended for CIED pocket infections [6,14-17]. For occult bacteremia, it is recommended to consult infectious disease and remove other sources of infection not related to the CIED. Without another identifiable source of infection, recommendations for CIED removal depend on the isolated organism, see Table 1 [14].

**Table 1:** Recommendations for CIED removal based on isolated organism CIED, cardiac implantable electronic device

Device removal	Observation w/o removal	Removal or observation
Staphylococcus aureus	Gram-negative bacteremia	Streptococcus species
Coagulase-negative Staphylococcus	Pneumococci	Enterococcus species
Propionibacterium		
Candida species		

In our case, extraction was indicated at the time of presentation due to pocket infection with MSSA bacteremia, as well as two months earlier due to occult MSSA bacteremia, in accordance with the aforementioned recommendations. When extraction is indicated, CIED removal can result in a substantial difference in outcomes relative to antibiotic therapy alone [8,18]. According to a retrospective study at the Mayo Clinic Rochester from 1991 to 2008, antibiotic therapy without extraction of CIED (when indicated) led to both a seven times greater 30-day mortality and an approximately three times greater one-year mortality [8,17]. While our patient had a favorable outcome, the case highlights the importance of CIED extraction in the setting of infection.

Multiple studies highlight the importance of timing in CIED extractions in relationship to outcomes. Early extraction has been associated with lower in-hospital mortality, mortality at 30 days, and mortality at one year relative to that of late extraction [7,8,10,18]. In addition to improving health outcomes, CIED extraction, particularly early extraction, has been shown to have economic benefits of shorter hospital stays and reduced overall costs within one year after the infection [8].

In our case, extraction was performed the day following this particular admission, which would be considered early. The patient had a short hospital stay and a benign post-operative course despite his age and comorbidities. However, it is worth noting that the patient presented two to three weeks after the onset of symptoms and had a history of bacteremia two months prior, which had been managed with antimicrobial therapy. Had this been managed according to the guidelines for occult bacteremia without an identifiable source, it may have prevented the second incidence of infection and the subsequent hospitalization. While we emphasize that early extraction is beneficial, we recognize that early extraction might not always be possible and can be limited by other considerations such as multiple comorbidities, frailty of patients, and extremes of age. We also recognize that the above-referenced studies may not have been fully inclusive of different population subgroups, practice settings, and other variables.

Another consideration for lead extraction is which type of extraction technique is used. Lead explant refers to the removal of a lead within one year of implantation using simple manual traction with a standard stylet and without the use of specialized tools. On the other hand, lead extraction refers to the use of specialized tools, removal using a different route from the implant vein, or removal of any lead more than one year after implantation [9]. Complex extraction techniques vary and involve the use of specialized extraction sheaths and/or other devices [9]. Simple extraction is generally thought to be effective for leads implanted within one to two years, with complex techniques being required for longer dwell times [9]. However, multiple studies have demonstrated successful simple extraction >2 years after lead implantation in >80% of patients, with major complication rates <2% [4,10]. Passive fixation and lead fracture can be more frequent with longer dwell times and have an impact on which approach is recommended [10]. In our patient’s case, lead implantation had been done less than 1 year before presentation, and a simple extraction was done successfully (Figure 2).

Another factor to consider is re-implantation versus no re-implantation, as redoing CIED procedures carries a higher risk of complications due to infection. Re-evaluation of the indication for CIED placement, along with a careful clinical assessment and device interrogation for dependency, is key to making that decision. Risk factors associated with infections should be reviewed. Patient-related factors, including age, symptomatology, comorbidities (e.g., renal failure, COPD, and diabetes mellitus), symptoms before and after insertion, underlying structural heart and electrical diseases, and medications (such as anticoagulants and corticosteroids), should be carefully considered. CIED interrogation for dependency can further support the decision on whether re-implantation is necessary. In our case, the decision not to re-implant was based on the patient’s comorbidities, lack of symptoms, and the additional confirmation that the patient was not pacemaker-dependent upon device interrogation [19].

## Conclusions

In conclusion, our case highlights a CIED pocket infection associated with MSSA bacteremia, successfully treated with early simple lead removal, resulting in a favorable outcome. Given the similar incidence of infection both early and late after implantation, it is crucial to remain vigilant regarding CIEDs as a potential source of infection and to consider removal when clinically indicated. Early recognition of signs of infections enables prompt action. It is also important to consider the method of lead removal when indicated and give consideration to simple extraction. As noted in the discussion, simple extraction has demonstrated favorable outcomes even more than two years after CIED insertion, despite generally being considered effective for devices implanted within one to two years. As CIED use continues to arise, we hope that this case highlights aspects of CIED infection and management that can benefit patient outcomes in terms of morbidity and mortality. In this case, there were no complications associated with simple extraction and the patient had a very brief hospital stay, a quick recovery, and a benign post-procedure course.
